# The prevalence and correlates of low resilience in patients prior to discharge from acute psychiatric units in Alberta, Canada

**DOI:** 10.1186/s12888-025-06704-8

**Published:** 2025-03-27

**Authors:** Ernest Owusu, Wanying Mao, Reham Shalaby, Hossam Eldin Elgendy, Belinda Agyapong, Ejemai Eboreime, Mobolaji A. lawal, Nnamdi Nkire, Yifeng Wei, Peter H. Silverstone, Pierre Chue, Xin-Min Li, Wesley Vuong, Arto Ohinmaa, Valerie Taylor, Carla T. Hilario, Andrew J. Greenshaw, Vincent I. O. Agyapong

**Affiliations:** 1 Department of Psychiatry, University of Alberta, Edmonton, AB Canada; 2 Department of Psychiatry, Dalhousie University, Halifax, NS Canada; 3 Alberta Health Services, Addiction and Mental Health Services, Edmonton, AB Canada; 4 School of Public Health, University of Alberta, Edmonton, AB Canada; 5 Department of Psychiatry, Cumming School of Medicine, University of Calgary, Calgary, AB Canada; 6 School of Nursing, University of British Columbia, Okanagan, BC Canada

**Keywords:** Discharge, Acute care, Mental Health, Resilience, Brief resilience scale

## Abstract

**Background:**

Many people experience at least one traumatic event in their lifetime. Although such traumatic events can precipitate psychiatric disorders, many individuals exhibit high resilience by adapting to such events with little disruption or may recover their baseline level of functioning after a transient symptomatic period. Low levels of resilience are under-explored, and this study investigates the prevalence and correlates of low resilience in patients before discharge from psychiatric acute care facilities.

**Methods:**

Respondents for this study were recruited from nine psychiatric in-patient units across Alberta. Demographic and clinical information were collected via a REDCap online survey. The brief resilience scale (BRS) was used to measure levels of resilience where a score of less than 3.0 was indicative of low resilience. A chi-square analysis followed by a binary logistic regression model was employed to identify significant predictors of low resilience.

**Results:**

A total of 1,004 individuals took part in this study. Of these 35.9% were less than 25 years old, 34.7% were above 40 years old, 54.8% were female, and 62.3% self-identified as Caucasian. The prevalence of low resilience in the study cohort was 55.3%. Respondents who identified as females were one and a half times more likely to show low resilience (OR = 1.564; 95% C.I. = 1.79–2.10), while individuals with ‘other gender’ identity were three and a half times more likely to evidence low resilience (OR = 3.646; 95% C.I. = 1.36–9.71) compared to males. Similarly, Caucasians were two and one-and-a-half times respectively more likely to present with low resilience compared with respondents who identified as Black (OR = 2.21; 95% C.I. = 1.45–3.70) or Asian (OR = 1.589; 95% C.I. = 1.45–2.44). Additionally, individuals with a diagnosis of depression were significantly more likely to have low resilience than those with a diagnosis of either bipolar disorder (OR = 2.567; 95% C.I. = 1.72–3.85) or schizophrenia (OR = 4.081;95% C.I. = 2.63–6.25).

**Conclusion:**

Several demographic and clinical factors were identified as predictors of likely low resilience. The findings may facilitate the identification of vulnerable groups to enable their increased access to support programs that may enhance resilience.

**Clinical trial registration:**

clinicaltrials.gov, NCT05133726. Registered on the 24th of November 2021.

## Introduction

Many people experience at least one traumatic event in their lifetime [1]. Although such traumatic events can precipitate psychiatric disorders, many people exhibit high resilience by adapting to such events with little disruption or may recover their baseline level of functioning after a transient symptomatic period [2, 3]. Psychological resilience is the capacity to adapt to adverse life events, the ability to cope with stress, and the ability to ‘bounce back’ after adversity [4, 5]. Resilience is essential in maintaining the quality of life, emotional well-being, and functional independence even if experiencing conditions as mental health challenges [6]. Many personal and environmental factors can impact resilience including personal and demographic characteristics, the level of external support, and social factors such as family cohesion [4, 7, 8]. Other factors can include psychological state, having a well-defined purpose in life, close friendships, the degree of religious belief, and other factors, such as gratitude, hope, and optimism [8].

One issue of note in terms of resiliency is gender, and vulnerability to trauma and stress differs between genders with resiliency overall being lower in women [9–11]. There have been some interventions which have increased this including increasing a women’s access to services, information, financial support, and employment and income-generating opportunities [12, 13]. Another factor in which resiliency can impact outcomes is mental health, including post-traumatic stress disorder (PTSD), depression, eating disorders, anxiety, bipolar disorder, and substance use disorders [11–14].

In terms of protective factors, high resilience appears to be a protective factor against the development of mental disorders after an adverse event [15]. For these reasons, it is possible that after an individual has a mental health issue, increasing their resilience by treatment including therapy may be beneficial in the management and recovery from their illnesses [16–18].

For these reasons it is important to understand the level of a patient resilience at the time of discharge and determine the effect of this, particularly since the transition from inpatient care into the community is often considered very difficult because of challenges relating to adjustment [19]. Many patients with mental health and emotional issues usually see the transition period as a test of their resilience, a threat to their recovery, and most often, some doubt their ability to cope with the everyday challenges that may confront them in the community [20]. Other areas of concern for these patients are their ability to deal with housing, work-related stress, job security, income support, and family support [21]. The thoughts of these and many other challenges frequently cause some patients to relapse even a few days before discharge [22]. It is possible that resilience levels may impact an individual’s ability to address their needs and fears prior to discharge [22], particularly since low resilience levels can reduce the patient's ability to deal with these concerns [23]. However, while there have been several studies demonstrating the importance of resiliency in discharge planning [24], there have been very few which have examined this in patients being discharged from mental health units.

The goal of the present work is to help bridge the current knowledge gap in which factors predict low resilience in patients prior to their discharge from acute mental health care setting. The specific objectives for this paper are:

- To evaluate the prevalence of low resilience for patients prior to discharge from inpatient psychiatric units in Alberta as measured by the Brief Resilience Scale (BRS) [25].
- To determine which clinical and sociodemographic variables may help predict low resilience among patients before discharge from acute psychiatric care settings.

**Hypothesis**

- We hypothesize that the prevalence of low resilience in the study cohort will be increased, and that this may be influenced by both demographic factors such as gender, age, ethnicity, and clinical factors including mental health diagnosis.

## Methodology

### Study setting and design

This study was conducted in the Canadian province of Alberta. Based on Government of Alberta statistics from July 1, 2023, Alberta has 4.7 million people residing in the province [26]. Nine acute mental health units across Edmonton, Calgary, and Grand Prairie in Alberta were the main sites [27]. The data in this study were collected as a part of a large ongoing pragmatic stepped-wedge cluster-randomized, longitudinal trial, fully detailed elsewhere [27]. In brief, this larger study aims to evaluate the impact of supportive text messages (Text4Support) and peer support services (PSS) on patients with mental illness after discharge from acute mental health facilities in Alberta. Study participants were recruited across nine main sites in Alberta as clustered randomization units [27]. The stepped wedge approach was deployed in four clusters over three years, with interventions spread over quarters within a year. This project was launched in March 2022, and follow-up data collection for the study is ongoing. The current study is based solely on the baseline data for the main study.

### Sample size calculation

With a margin of error of + 3%, an estimated inpatient population of 28,571, and a confidence interval of 95%, we estimated, using an online app [28], that the sample size needed to assess the prevalence of low resilience would be 829.

### Data collection

The data for this study was collected through RedCap [29], an online data collection platform. Eligibility criteria were a service user, aged 18 and above, diagnosed with any mental condition, ready to be discharged from an inpatient mental health unit, had a mobile device, could read English text messages, and could provide informed written consent.

Sociodemographic information included age, gender identity, ethnicity, educational level, relationship status, and employment status. Clinical information such as diagnosis and duration of the service user's admission were also collected. Recruitment occurred between March 1, 2022, and November 5, 2023. All study participants completed the baseline online survey with the facilitation of research team members after signing a paper-based consent form. Phone numbers and healthcare numbers were considered primary identifiers for the service users, and the phone numbers were used to track the responses across the follow-up time points.

### Outcome measures

A main outcome measure of interest is the prevalence of low resilience. Other outcomes of interest include the relationship between gender, age, ethnicity, and mental health diagnosis against low resilience.

The BRS is a self-assessment tool used to evaluate resilience, with the title of the primary article being “*The brief resilience scale: assessing the ability to bounce back”* [5, 25, 30]. The scale consists of six statements for which an individual is expected to state to what extent they agree or disagree with these statements, and these statements incorporate both positively and negatively worded items [31]. The total score is calculated by adding the responses (varying from 1–5) for each of the six items on a Likert scale, yielding a total score ranging from 6–30. The BRS has an acceptable internal consistency reliability (*a* = 0.66) and test–retest reliability (*r* = 0.67) [31]. A final score in the BRS is determined by dividing the total score by the total number of answered questions. Higher scores indicated higher levels of resilience. Past work has shown that scores indicating low resilience are from 1.00–2.99, scores from 3.00–4.30 indicate normal levels of resilience; and scores from 4.31–5.00 indicate high levels of resilience [5, 25, 31]. In the present analysis, we combined scores in the normal and high resilient ranges into one category, i.e. those scores that were between 3.00–5.00 and compared these to those who scored in the low resilience range (i.e. 1.00–2.99), which are considered vital psychometric properties of the BRS scale.

### Statistical analysis

Data analysis for this study was performed using SPSS for Mac, version 25 (IBM et al., USA) [32]. Descriptive statistics focused on the respondents’ sociodemographic and clinical characteristics against ethnicity status. A Univariate analysis using Chi-square was performed to examine all the variables’ association with the likely low resilience categorical variable (normal to high and low resilience). The significant (p ≤ 0.05) or near significant (0.1 > *p* > 0.05) variables obtained from the univariate analysis were included in a binary logistic regression model. Also, a correlational analysis was used to rule out strong inter-correlations (Spearman’s correlation coefficient of 0.7 to 1.0 or − 0.7 to − 1.0) between the variables. The logistic regression model was employed to identify significant predictors of likely low resilience. Confidence intervals and odds ratios (OR) were used to determine the predictor variables for respondents to self-report with low resilience before discharge.

## Results

Table 1 depicts the distribution of participants' sociodemographic and clinical characteristics compared to self-identified ethnicity. The majority self-identified as Caucasian, (62.3%); while some self-identified as Indigenous (9.5%); as Black (10.2%); as Asian (11.1%); and as ‘Others’ (6.9%). Out of the 1,004 participants, 35.9% were less than 25 years old, and 34.7% were above 40 years old. The majority of participants identified as female (54.8%), had a high school diploma (51.4%), were single ( 58.9%), unemployed (53.4%), and the largest group lived in rented homes (32.3%). In terms of clinical status, the largest diagnostic group had a diagnosis of depression (26.1%).

**Table 1:** Demographic and clinical information on the participants

**Variables**	Caucasians **N (%)**	Indigenous People **N (%)**	Black People **N (%)**	Asians N (%)	Other **N (%)**	**Total** **N (%)**
**Age categories (**years)						
≤ 25 years	191 (30.6)	39 (41.1)	52 (50.5)	55 (49.9)	23 (32.9)	360 (35.9)
26–40 years	221 (35.4)	28 (29.5)	37 (35.9)	34 (30.6)	28 (40.0)	348 (34.7)
**> **40 years	213 (34.1)	28 (29.5)	14 (13.6)	22 (19.8)	22 (27.1)	296 (29.5)
Total	625 (100)	95 (100)	103 (100)	111 (100)	70 (100)	1004 (100)
**Gender**						
Male	261 (41.8)	31 (32.6)	47 (45.6)	48 (43.2)	39 (55.7)	425 (42.4)
Female	345 (55.2)	60 (63.2)	55 (53.4)	61 (55.0)	29 (41.4)	550 (54.8)
Other Gender	19 (3.0)	4 (4.2)	1 (1.0)	2 (1.0)	2 (2.9)	28 (2.8)
Total	625 (100)	95 (100)	103 (100)	111 (100)	70 (100)	1004 (100)
**Education categories**						
Less than High School	27 (4.3)	3 (3.2)	3 (2.9)	3 (2.7)	3 (4.3)	39 (3.9)
High School Diploma	301 (48.2)	64 (67.4)	58 (56.3)	56 (50.5)	37 (52.9)	516 (51.4)
Post-Secondary Education	277 (44.3)	25 (26.3)	36 (35.0)	50 (45.0)	29 (41.4)	417 (41.5)
Other	20 (3.2)	3 (3.2)	6 (5.8)	2 (1.8)	1 (1.4)	32 (3.2)
Total	625 (100)	95 (100)	103 (100)	111 (100)	70 (100)	1004 (100)
**Current Relationship Status**						
Single	338 (54.1)	64 (67.4)	76 (73.8)	71 (64.0)	42 (60.0)	591 (58.9)
Separated /Divorced	60 (9.6)	4 (4.2)	4 (3.9)	5 (4.5)	7 (10.0)	80 (8.0)
Partnered/Married/Common law	199 (31.8)	23 (24.2)	19 (18.4)	34 (30.6)	17 (24.3)	292 (29.1)
Widowed	7 (1.1)	1 (1.1)	1 (1.0)	0 (0.0)	1(1.4)	10 (1.0)
Other	21 (3.4)	3 (3.2)	3 (2.9)	1 (0.9)	3 (4.3)	31 (3.1)
**Total**	625 (100)	95 (100)	103 (100)	111 (100)	70 (100)	1004 (100)
**Current Employment Status**						
Student	37 (5.6)	4 (4.2)	7 (6.8)	24 (21.6)	5 (7.1)	77 (7.7)
Employed	198 (31.7)	19 (20.0)	25 (24.3)	29 (26.1)	27 (38.6)	298 (29.7)
Unemployed	321 (51.4)	69 (72.6)	65 (63.1)	52 (46.8)	29 (41.4)	536 (53.4)
Retired	47 (7.5)	0 (0.0)	3 (2.9)	3 (2.7)	5 (7.1)	58 (5.8)
Other	22 (3.5)	3 (3.2)	3 (2.9)	3 (2.7)	4 (5.7)	35 (3.5)
**Total**	625 (100)	95 (100)	103 (100)	111 (100)	70 (100)	1004 (100)
**Current Housing Status**						
Own Home	163 (25.9)	10 (10.5)	7 (6.8)	19 (17.1)	7 (10.0)	205 (20.4)
Rented Accommodation	209 (33.4)	42 (44.2)	23 (22.3)	18 (16.2)	32 (45.7)	324 (32.3)
Live with Family/Friends	218 (34.9)	36 (37.9)	63 (61.2)	69 (62.2)	26 (37.1)	412 (41.0)
Couch/Shelter/Street/Other	36 (5.8)	7 (7.4)	10 (9.7)	5 (4.5)	6 (7.1)	63 (6.3)
**Total**	625 (100)	95 (100)	103 (100)	111 (100)	70 (100)	1004 (100)
**Primary MH Diagnosis (Dx)**						
Depression	169 (27.0)	30 (31.6)	19 (18.4)	28 (25.2)	18 (22.9)	262 (26.1)
Bipolar Disorder	138 (22.1)	11 (11.6)	18 (17.5)	22 (19.8)	17 (24.3)	206 (20.1)
Anxiety Disorder	86 (13.8)	7 (7.4)	17 (16.5)	19 (17.1)	6 (8.6)	135 (13.2)
Schizophrenia	76 (12.2)	23 (24.2)	28 (27.2)	18 (16.2)	16 (22.9)	161 (16.0)
Personality Disorder	60 (9.6)	9 (9.5)	4 (3.9)	11 (9.9)	7 (10.0)	91 (9.1)
Substance Use Disorder	27 (4.3)	8 (8.4)	6 (5.8)	8 (7.2)	2 (2.9)	51 (5.1)
Other	69 (11.0)	7 (7.4)	11 (10.7)	5 (4.5)	6 (8.6)	98 (9.8)
**Total**	625 (100)	95 (100)	103 (100)	111 (100)	70 (100)	1004 (100)

A chi-square test determined the association between demographic and clinical factors and resilience (Table 2). Gender category (Χ2 (2) = 30.2; *p* < 0.001), ethnic category (Χ2 (4) = 35.7; *p* < 0.001), and current housing status (Χ2 (3) = 8.8; *p* < 0.05) each had an association with low resilience. Also, MH Dx (Χ2(6) = 81.9; *p* < 0.001) had an association with low resilience.

**Table 2:** Chi-squared association test between the demographic and clinical antecedents and likely low resilience

Variables	Normal to high resilience	Low Resilience	Chi-square	*P*-value
**Demographic Characteristics**				
**Age categories (years)**				
≤ 25 years	143 (39.7%)	217 (60.3%)	5.68	.058
26–40 years	166 (47.7%)	182 (52.3%)		
**> **40 years	140 (47.3%)	156 (52.7%)		
**Gender**				
Male	231 (54.2%)	195 (45.8%)	30.2	.001
Female	212 (38.5%)	338 (61.5%)		
Other Gender	6 (21.4%)	22 (78.6%)		
**Ethnicity Categories**				
Caucasian	242 (38.7%)	383 (61.3%)	35.7	.001
Indigenous	44 (46.3%)	51 (53.7%)		
Black	62 (60.2%)	41 (39.8%)		
Asian	53 (47.7%)	58 (52.3%)		
Other	48 (68.6%)	22 (31.4%)		
**Education Categories**				
Less than High School	17 (43.6%)	22 (56.4%)	3.7	.298
High School Diploma	225 (43.6%)	291 (56.4%)		
Post-Secondary Education	197 (47.2%)	220 (52.8%)		
Other	10 (31.3%)	22 (68.8%)		
**Current Relationship Status**				
Single	276 (46.7%)	315 (53.3%)	9.0	.059
Separated /Divorced	36 (45.0%)	44 (55.2%)		
Partnered/Married/Common law	122 (41.8%)	170 (58.2%)		
Widowed	7 (70.0%)	3 (30.0%)		
Other	8 (25.8%)	23 (74.2%)		
**Current Employment Status**				
Student	30 (39.0%)	47 (61.0%)	4.4	.350
Employed	145 (48.7%)	153 (51.3%)		
Unemployed	231 (43.1%)	305 (56.9%)		
Retired	29 (50.0%)	29 (50.0%)		
Other	14 (40.0%)	21 (60.0%)		
**Current Housing Status**				
Own Home	88 (42.9%)	117 (57.1%)	8.8	.031
Rented Accommodation	155 (47.8%)	169 (52.2%)		
Live with Family/Friends	169 (41.0%)	243 (59.0%)		
Couch/Shelter/Street/Other	37 (58.7%)	26 (41.3%)		
**Primary MH Diagnosis**				
Depression	80 (30.5%)	182 (69.5%)	81.9	.001
Bipolar Disorder	107 (51.9%)	99 (49.1%)		
Anxiety Disorder	53 (39.3%)	82 (60.7%)		
Schizophrenia	108 (67.1%)	53 (32.9%)		
Personality Disorder	26 (28.6%)	65 (71.4%)		
Substance Use Disorder	35 (68.6%)	16 (31.4%)		
Other	40 (40.8%)	58 (59.2%)		

Table 3, as illustrated, used the binary logistic regression model to predict likely low resilience among respondents. The model included all four variables that showed a significant association with low resilience and two others that showed a nearing significant association (Age, *p* = 0.06, and current relationship status, *p* = 0.06) on Chi-Square analysis. No high inter-correlated variables were detected (rs > 0.7), and all six variables were included in the regression model.

**Table 3:** Multivariate logistic regression model that predicts the likely low resilience among participants

Characteristics	B	S.E	Wald	df	*P* value	Odd’s Ratio	95% C.I. for Odd’s Ratio	
							Lower	Upper
**Age categories (years)**								
≤ 25 years			1.079	2	.583			
26–40 years	-.130	.177	.542	1	.462	.878	.621	1.241
**> **40 years	-.219	.214	1.040	1	.308	.804	.528	1.223
**Gender**								
Male			14.091	2	< .001			
Female	.447	.144	9.609	1	.002	1.564	1.179	2.074
Other	1.294	.500	6.701	1	.010	3.646	1.369	9.711
**Ethnicity Categories**								
Caucasians			27.940	4	< .001			
Indigenous People	-.306	.240	1.619	1	.203	.737	.460	1.180
Black People	-.837	.238	12.364	1	< .001	.433	.271	.690
Asians	-.464	.224	4.286	1	.038	.629	.405	.976
Other	−1.197	.286	17.541	1	< .001	.302	.173	.529
**Current Relationship Status**								
Married			3.495	4	.479			
Separated	.169	.276	.375	1	.540	1.184	.689	2.034
Partnered/Married/Common law	.061	.173	.125	1	.724	1.063	.758	1.491
Widowed	-.995	.746	1.776	1	.183	.370	.086	1.597
Other	.475	.451	1.108	1	.293	1.608	.664	3.894
**Current Housing Status**								
Own Home			6.610	3	.085			
Rented accommodation	-.111	.205	.291	1	.589	.895	.599	1.338
Live with family/ friends	.269	.227	1.403	1	.236	1.309	.839	2.042
Couch/Shelter/street/other	-.346	.329	1.109	1	.292	.707	.371	1.348
**Primary MH Diagnosis**								
Depression			56.052	6	< .001			
Bipolar disorder	-.940	.201	21.819	1	< .001	.391	.263	.580
Anxiety disorder	-.431	.231	3.498	1	.061	.650	.414	1.021
Schizophrenia	−1.408	.227	38.472	1	< .001	.245	.157	.382
Personality disorder	-.162	.288	.315	1	.574	.851	.484	1.496
Substance Use Disorder	−1.477	.340	18.876	1	< .001	.228	.117	.444
Other	-.462	.259	3.186	1	.074	.630	.379	1.046
Constant	-.065	.256	.064	1	.800	.937		

The model was statistically significant; Χ2 (df = 12; *n* = 164) = 65.60, *p* < 0.001, indicating that the model could differentiate between respondents who did or did not exhibit likely normal to high or low resilience before discharge from the hospital. The model accounted for 13.2.0% (Cox and Snell R2) to 17.6% (Nagelkerke R2) of the variance and accurately classified as 66.8%.

Three main variables, namely gender, ethnicity, and mental health diagnosis, could significantly predict the low resilience among study respondents. Respondents who identified as females were one and a half times more likely to evidence low resilience (OR = 1.564; 95% C.I. = 1.79–2.10), while other genders were about three and half times more likely to exhibit low resilience (OR = 3.646; 95% C.I. = 1.36–9.71) compared to male gender when controlling for other variables. Caucasian participants were two times more likely to present with likely low resilience compared with Black people (OR = 2.21; 95% C.I. = 1.45, 3.70), almost two times compared with Asians (OR = 1.589; 95% C.I. = 1.45–2.44) and three times compared with ‘Other’ ethnicity (OR = 3.311; 95% C.I. = 1.89–5.88).

Similarly, persons with the diagnosis of depression were more likely to present with low resilience by more than two times as compared to those with bipolar disorder (OR = 2.567; 95% C.I. = 1.72–3.85), four times as compared to both those having Schizophrenia (OR = 4.081;95% C.I. = 2.63–6.25) or SUD (OR = 4.385; 95% C.I. = 2.27–7.69) after controlling for all other variables in the model.

## Discussion

The prevalence of low resilience in this cohort was 55.3%. In terms of comparative research, most articles on resilience do not examine this in mental health populations. The few that do show suggest lower rates of low resilience that we found, for example studies carried out during the COVID-19 pandemic (36.4% and 24.1%) [33]. Such low levels of resilience in the current cohort may indicate that a significant portion of the population may struggle to cope with subsequent stress, adversity, and life challenges post-discharge. This has implications for increased rates of mental health challenges, depression, anxiety, and other stress-related conditions.

In terms of potential risk factors, which may need specific recognition in the future, the present results indicated that respondents who were females, and those who identified as ‘other’ genders, were almost two times and four times, respectively, more likely to develop low resilience than respondents who identified as males. These findings are consistent with previous studies which have found that males generally have higher levels of resilience [10, 34]. This also aligns with previous research indicating that male students exhibit higher resilience, particularly in areas such as personal power, initiative, foresight, purpose in life, leadership, and investigative skills [35]. In contrast, other factors did not appear to significantly impact levels of low resilience, including age, housing, and relationship status.

One factor that did impact levels of low resilience was ethnicity. Those who identified as Caucasian were two times more likely to present with low resilience compared with participants who identified as Black or Asian, and approximately three times more likely to have low resilience compared with those who identified their ethnicity as ‘Other’. This finding adds to past results which have investigated mental health disparities among Canadians based on race and ethnicity, and have found that different ethnic groups exhibited difference in mental health conditions [36–39], although these past studies did not examine resilience specifically. Since it is recognized that high resilience may improve clinical outcomes [38], it is possible that these two factors may be related and further research to explore the possible role that ethnicity may have in outcomes post-discharge is warranted. Factors such as cultural identity and community connections may play a crucial role in fostering resilience and providing individuals with the support they need to overcome challenges and enhance their overall well-being. The outcome of a study examining the quality of life about the role of culture-specific coping as related to resilient outcomes in African Americans from high-risk urban communities found that family cohesiveness, a common feature in African Americans, to be a traditional protective factor [40].

There were also differences in terms of diagnosis, with individuals with a diagnosis of depression being more likely to present with low resilience compared with those with bipolar disorder, schizophrenia, or substance use disorder. This finding aligned with past results from a study that explored the relationship between resilience and clinical measures in patients diagnosed with severe mental disorders in an inpatient setting [41]. The potential role for intervention is supported by a study which found that social work interventions which are focused on enhancing resilience among persons with substance use disorder can be helpful in maintaining long-term abstinence [42].

### Limitations

The study is conducted not without limitations. One notable limitation is the absence of a precise control group. The results may not be as widely applicable or generalizable to other populations or settings. A control group would have allowed for better comparisons, helping to determine whether the factors being studied had a significant impact compared to a non-intervention group.

Another limitation of the study is the potential for selection bias. To be included in the study, participants were required to have a cell phone with an active line to receive the intervention. This criterion meant that individuals without active cell phones were excluded from participation. As a result, we could not gather clinical data, including resilience levels, from these excluded individuals. This introduces a bias because the sample may not fully represent the broader population of patients in acute mental health facilities, potentially affecting the study outcome.

Additionally, while the study collected data on the gender of the participants, it did not gather information about the participants' biological sex. This omission means we could not explore whether biological sex could influence the study's outcomes. Moreover, we also did not gather data on individuals whose sex at birth differed from their identified gender, which could have provided insight into how gender identity may impact resilience levels. As such, this lack of detailed demographic information limits our understanding of how gender and biological sex might interact with resilience.

Finally, another limitation is that the study did not establish the relationship between other forms of positive adaptation, such as thriving and the Brief Resilience Scale [43]. Understanding whether thriving is related to resilience could have added a deeper layer of understanding to our findings. The absence of this exploration means that we missed an opportunity to link other forms of positive psychological outcomes to resilience potentially.

Despite these limitations, the study offers valuable insights into the prevalence and predictors of low resilience in patients with mental health challenges who are on admission to various acute mental health facilities in Alberta. The size of the study and the breadth of its findings still contribute significantly to understanding the factors influencing resilience in this population.

## Conclusions

In conclusion, the study found that individuals in this cohort generally exhibited low levels of resilience. We identified several demographic and clinical factors, including gender, ethnicity, and primary diagnosis, as predictors of low resilience. Since low resilience has been linked to poorer clinical outcomes in previous research, our findings highlight the importance of assessing resilience in individuals admitted for mental health care. Given that resilience can be improved through various interventions, addressing this factor presents an opportunity for better long-term outcomes after discharge. Specifically, females and individuals from certain ethnic groups appeared at higher risk for low resilience, suggesting that these populations may benefit from targeted interventions. Policymakers should consider prioritizing funding for programs aimed at enhancing resilience in vulnerable groups, including women, young adults, and Indigenous communities. Evidence-based interventions such as daily supportive text messaging [44, 45] and peer support services [46, 47], which have shown effectiveness in improving mental health, could play a vital role in fostering resilience. Additionally, addressing social determinants of mental health may inform targeted policies that enhance resilience at the individual, community, and societal levels, contributing to improved long-term mental health outcomes.

## Data Availability

The data in this study are not publicly available due to ethical and privacy reasons, but can made available on request from the corresponding author.
